# The Effect of Time Pressure on the Quality of Fillings and Arousal Levels of Dentists

**DOI:** 10.1002/cre2.70106

**Published:** 2025-02-24

**Authors:** Kaisa M. Leinonen, Jukka Leinonen, Mohammed Al‐Haroni, Jan‐Are K. Johnsen

**Affiliations:** ^1^ Department of Clinical Dentistry, Faculty of Health Sciences UiT The Arctic University of Norway Tromsø Norway; ^2^ Institute of Dentistry, School of Medicine University of Eastern Finland Kuopio Finland

**Keywords:** arousal, composite resin, experience, time pressure

## Abstract

**Objectives:**

We investigated the impact of time pressure on dentists' arousal levels and the immediate quality of composite resin fillings using two filling techniques in a simulated clinical setting.

**Material and Methods:**

Forty‐two dentists and dental students were randomized to fill an identical Class II cavity either with the bulk‐fill base technique or the conventional incremental technique. The task was performed first under timing and then under time pressure. Arousal levels were investigated with a 100 mm visual analog scale (VAS) and a wireless wrist‐mounted triaxial accelerometer. The surface porosity and marginal gaps on the approximal surfaces of the fillings were evaluated using a stereomicroscope and specific probes according to the FDI criteria for restoration evaluation.

**Results:**

Time pressure significantly increased arousal overall (*F* = 14.98, *p* < 0.05), and there was a significant effect of the experimental group on participants' arousal levels under time pressure (*F* = 7.78, *p* < 0.05); Dunn–Bonferroni tests indicated significantly lower arousal levels (*p* < 0.05) for the bulk‐fill base technique (*M* = 4.53, SD = 2.17) compared to the conventional incremental technique (*M* = 6.68, SD = 1.86). The bulk‐fill base technique showed superior quality under time pressure (X^2^ = 4.71, *p* = 0.030). Less experienced operators achieved better quality with the bulk‐fill base technique (Χ^2^ = 5.62, *p* = 0.018), while operator experience did not correlate with arousal levels under time pressure.

**Conclusions:**

Employing the bulk‐fill base technique under time pressure led to reduced operator arousal levels and improved the immediate quality of fillings.

**Clinical Relevance:**

The bulk‐fill base technique shows promise in being resistant to common work environment factors in dentistry, such as time pressure and stress, without compromising the quality of fillings. Utilizing innovative techniques, such as the bulk‐fill base technique, in a clinical setting can contribute to mitigating the operator's stress and enhancing the quality of care provided.

## 1 Introduction

One out of eight dentists have suffered burnout syndrome depicting the stressful nature of the profession (Moro et al. 2022). Time pressure is one of the biggest stressors for two‐thirds of dentists (Collin et al. 2019). Stress is a response to a physical or psychological stressor that induces arousal and can lead to either a positive or negative outcome depending on the intensity, repetition, and duration of the stressor (Mahoney and Chapman 2004; Chu et al. 2024). In psychology, arousal refers to a spectrum within emotions that indicates a person's degree of activation or alertness (Russell 2003). This spectrum spans from low arousal, characterized by feelings of relaxation, calmness, or boredom, to high arousal, which includes sensations such as nervousness, tension, or excitement (Russell 2003). Elevated levels of arousal often improve performance, but with too high levels of arousal cognitive impairment often appears (Mahoney and Chapman 2004; Chu et al. 2024). In response to cognitively demanding tasks, such as performing tasks in insufficient time, arousal levels elevate (King et al. 1983). The emotional state of the health care provider has been suggested to have a detrimental effect on clinical decision‐making, which can ultimately impact quality of care (Croskerry et al. 2010).

The most commonly used material for direct restorations today is resin‐based composite (Eltahlah et al. 2018; Leinonen et al. 2021). Dentists spend 58% of their working hours placing fillings (Staxrud et al. 2016), and of all fillings nearly 60% are re‐operations, either replacements or reparations (Eltahlah et al. 2018). Factors related to the patient and the operator seem to have a significant impact on restoration longevity, whereas the influence of adhesive strategies and restorative materials appears to be less pronounced (Schwendicke et al. 2020). Operator experience is often considered a key factor in restoration longevity; however, the scientific evidence on this remains inconsistent (Burke and Lucarotti 2018).

Two topical techniques to fill a cavity with resin‐based composite are the conventional incremental technique and the bulk‐fill base technique. The conventional incremental technique requires meticulous thin layering of several maximum 2 mm increments and is therefore technique‐sensitive and time‐consuming (Al‐Zain et al. 2023; Ferracane 2024; Sword et al. 2021; Leinonen et al. 2023). In contrast, specific bulk‐fill base composites enable using bulk‐fill base technique. It consists of applying a thick bottom layer, up to 4 or 5 mm, of typically flowable bulk‐fill composite and a capping layer of viscous composite (Ferracane 2024). Although wear resistance and polymerization shrinkage of newer bulk‐fill base materials are comparable to those of viscous materials (Turk et al. 2024; Khoramian Tusi et al. 2022). As a result of the ease‐of‐use of the bulk‐fill technique, it takes four and half minutes less time compared to the conventional incremental technique when filling a large class II cavity (Leinonen et al. 2023). In addition, the immediate quality of fillings performed with bulk‐fill base technique is better compared to the conventional incremental technique (Leinonen et al. 2023). The simplified application process of bulk‐fill resin‐based composites not only enhances efficiency but also decreases the likelihood of procedural errors associated with multiple layering steps (Al‐Zain et al. 2023; Leinonen et al. 2023). Bulk‐fill resin‐based composites are a safe alternative to conventional resin composites because they possess similar or better clinical performance and chemical‐physical properties (Khoramian Tusi et al. 2022; Silva et al. 2023; Schoilew et al. 2023). The reduction in procedural complexity of cavity filling using bulk‐fill base technique instead of the conventional incremental technique may alleviate stress and cognitive load for dentists.

Time pressure can increase cognitive load and feeling of uncontrollability, potentially forcing clinicians to multitask or prioritize speed over accuracy (ALQahtani et al. 2016; Plessas et al. 2019; Ehrhardt et al. 2022). This mental strain further amplifies arousal levels, which can lead to errors when arousal exceeds optimal levels (Cheng et al. 2017). Also, over‐arousal may lead to experiences of cognitive overload which can impair fine motor skills that are essential for procedures such as filling a cavity (Lazarus and Folkman 2009). Critical steps inherent to the conventional incremental technique may be rushed under time constraints, compromising the quality of the filling. In contrast, flowable bulk‐fill base composites settle easily and well into the cavity, eliminating the need to compact the material.

Given that time pressure could be argued to impact clinical decision‐making, it is interesting to examine how various clinical procedures might interact with arousal and time pressure (ALQahtani et al. 2016; Plessas et al. 2019). To the best of our knowledge, there are no studies on the effect of time pressure and arousal on the quality of the fillings (Plessas et al. 2018). The primary aim of this study was to determine the effect of time pressure on the arousal levels of dentists and dental students while filling a large Class II cavity. The secondary aim of our study was to compare the quality of the fillings placed under time pressure using the bulk‐fill base technique or the conventional incremental technique.

The following null hypothesis were set: (1) time pressure increases the stress level of the operator, (2) the presence of time pressure decreases the quality of fillings, (3) the presence of time pressure has less of an effect on the quality of fillings made using bulk‐fill base technique than using the conventional incremental technique, and (4) the presence of time pressure effects more the less experienced operators and the quality of their fillings.

## 2 Methods

The protocol for participant recruitment, laboratory settings, cavity dimensions, filling techniques and filling quality assessments have been described in detail earlier (Leinonen et al. 2023).

**Figure 1:**
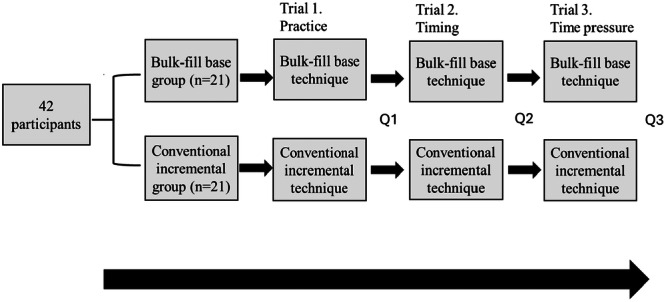
Study design. Q = questionnaire.

The experiment consisted of three trials. The participants were randomly allocated to either to the conventional incremental technique group or the bulk‐fill base technique group. The experimental group stayed the same through each trial. In the first trial, all participants placed a practice filling to familiarize themselves with the study environment. In the second trial, the time taken to fill the cavity was recorded in seconds using a handheld stopwatch. In the third trial, participants filled the cavity under time pressure (Figure 1). A countdown timer, displaying the remaining time for participants to complete the procedure, was clearly visible on a screen when the participants were performing the third trial. Work had to be halted when the timer reached zero.

The time constraint for the third trial was determined separately for both filling techniques based on a pilot study, which involved seven dentists with work experience ranging from less than 1 year to 10 years, performing the experiment using both techniques. The pilot study provided data to calculate the mean duration (TMp) and standard deviation (SDp) for each filling technique and determine the time constraint for each technique using the equation TP = TMp – SDp (Ordóñez and Benson 1997). The method for calculating time pressure using one standard deviation below the mean pretest time was first introduced by Svenson and Benson in 1993 (Svenson and Benson 1993a). Studies appling this novel metric to detemine time constraint have found that the time constraint compelled 84%–100% of subjects to perform the given task faster than normal (Ordóñez and Benson 1997; Benson and Beach 1996).

The time available was 68% for the conventional incremental technique and 78% for the bulk‐fill base technique, reflecting the relative efficiency and expected time pressure of each method. The time available for the third trial was calculated separately for each participant based on the time consumed in the second trial. For example, if a participant completed the second trial in 120 s using the bulk‐fill technique, the time available for the third trial would be 78% of 120 s, equating to 93.6 s. Time constraints alone are not sufficient to ensure that subjects experience time pressure; however, if the time constraint induces a sense of stress/arousal, it may indicate time pressure (Svenson and Benson 1993b).

Arousal levels were investigated using questionnaires and accelerometer data on hand movements. These movements can be reliably quantified through triaxial accelerometers, which measure g‐forces along the orthogonal X, Y, and Z axes to capture comprehensive movement dynamics (Yuan et al. 2023). Accelerometers are generally not used for detecting emotions but rather for measuring movement and orientation; they explicitly output the forces exerted on the device (Prasad et al. 2021). However, prior studies have shown that accelerometer data can detect emotional activation and that during arousing conditions, both relevant and irrelevant hand movements increase significantly (Bruns Alonso et al. 2007; Piskioulis et al. 2021; Saddaf Khan et al. 2024).

The participants wore a wireless wrist‐mounted triaxial accelerometer (BioNomadix Tri‐Axial Accelerometer, BN‐ACCL3 Receiver+Transmitter and BIOPAC MP150 unit, AcqKnowledge 4.4.2 Software, BIOPAC Systems Inc., Goleta, California) on their dominant hand to measure the acceleration of hand movements. The accelerometer operates within a ±16 g limit and provides output corresponding to the orthogonal X, Y, and Z axes, enabling a comprehensive assessment of the dynamics of hand movements across these dimensions. The wireless accelerometer was connected to a Biopac MP150 unit through the BioNomadix ACCL3‐R wireless expansion module, and data was registered and analyzed by AcqKnowledge software. To explore how hand movements were affected by the experimental conditions, the start and end points of each experimental trial were marked in the experimental software package, which allowed the mean g‐force/acceleration to be determined for each trial. Sampling frequency was set to 2.0 kHz to enable precise registration of minor hand movements (Samson et al. 2001). The start and end points for each trial were recorded to determine the mean acceleration separately for each trial. This method complements questionnaire measures and offers a novel way to detect fluctuations in arousal, as traditional self‐report measures may be subjective or prone to recall biases (Choi and Pak 2005).

After each of the three trials, the participants registered their self‐reported levels of arousal, using a visual analog scale (VAS) consisting of a 10‐cm line. As end points on the VAS, two adjective pairs from the Short Adjective Check List (SACL) were used which the participants used to indicate their levels of arousal (0 cm = tense – 10 cm = relaxed; 0 cm = nervous – 10 cm = calm) (Mackay et al. 1978). The VAS scores from both SACL items were combined into one mean arousal score. To clarify data interpretation, arousal scores were reversed from the original scoring scheme, so that high scores indicate high arousal.

### 2.1 Ethics

The protocol for this study was approved by the Norwegian Centre for Research Data (reference number 399613). The protocol was reviewed by the Regional Committees for Medical and Health Research Ethics which concluded that the study did not qualify as health research and therefore did not require their approval (reference number 158600).

### 2.2 Statistics

The Shapiro–Wilk test revealed that the data on arousal was normally distributed. The repeated measures of ANOVA were performed to explore if there was a main effect of the experiment on arousal levels of the participants, and if the experimental group had an effect on the arousal levels. To explore the effects of time pressure in more detail, Dunn–Bonferroni post‐hoc tests were performed to see how arousal varied across trials. Student's *t‐*test was used to compare the arousal between and within the bulk‐fill base and the conventional incremental groups. One‐way ANOVA was performed to compare whether the usual filling technique of choice was associated with arousal levels with and without time pressure.

The Chi‐square test of independence was performed to examine the differences in surface porosity and marginal gaps for the filling technique groups and different levels of clinical experience. The Chi‐square test was also used to compare whether the usual filling technique of choice was associated with the quality of the filling under time pressure. The Chi‐square test was also performed to explore the overall effect of the experiment on the quality of the fillings.

The Shapiro–Wilk test revealed that the accelerometer data was not normally distributed. The Mann–Whitney *U* test was used to compare G‐force between the experimental groups. Spearman's rank order correlation test was used to check the strength and the direction of association between stress and accelerometer data.

All analyses were performed with SPSS version 29 (IBM SPSS Statistics) and JASP 0.16.1. The significance level was set at *p* < 0.05.

## 3 Results

Among the participants, there were 18 dentists (83.3% female) and 24 dental students (75.0% female). Before the study, the student participants had placed an average of 126.6 fillings (SD = 63.7, range = 30–275).

The conventional incremental technique was reported as the usual technique to fill a Class II cavity by 46.3% of participants, whereas 48.8% of the participants reported they usually use the bulk‐fill base technique. Among the dental students, the bulk‐fill base technique was the usual choice for 56.5%, and the conventional incremental technique for 39.1%. Conversely, among the dentists, the bulk‐fill base technique was the usual choice for only 38.8% and the conventional incremental technique for 55.5%. The reported usual filling technique of choice was not associated with the arousal levels of the operator or the quality of the fillings with or without time pressure.

Hypothesis 1 Time pressure increases the arousal level of the operator.

**Figure 2:**
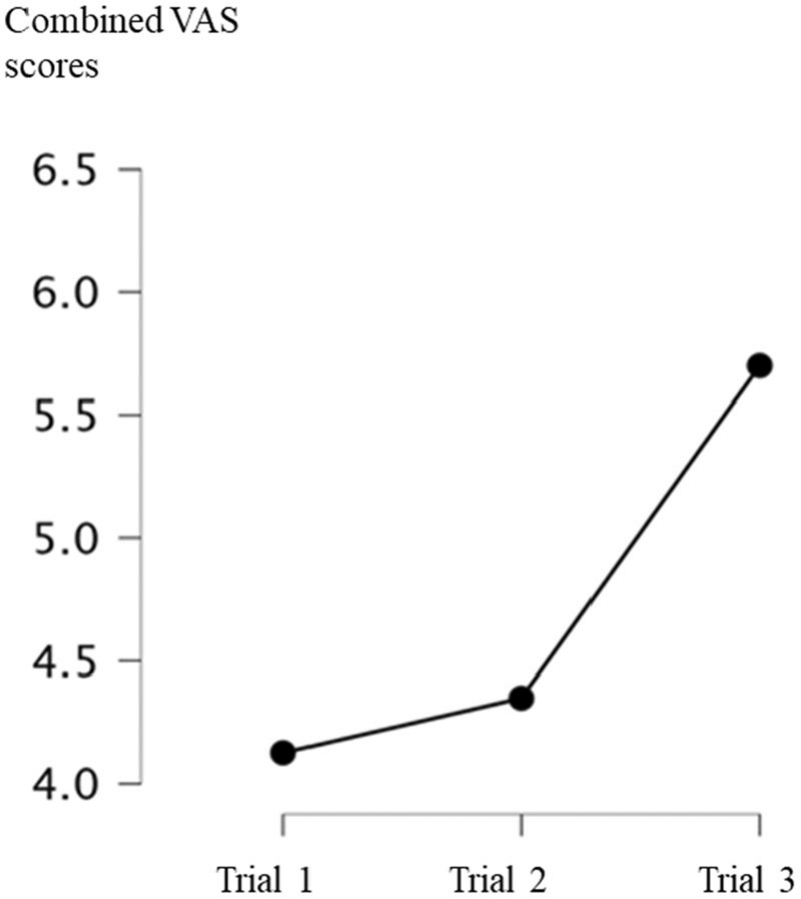
The overall effect of the experiment on the arousal levels. The participant's self‐reported arousal levels (means) using VAS across conditions (plots) and trials (*x* axis).

There was a significant main effect of the whole experiment on self‐reported arousal levels, in which the participants reported higher arousal levels as the experiment progressed (df between subject effects = 1.44, *F* = 14.98, *p* < 0.05, Figure 2).

To explore the effects of time pressure in more detail, post‐hoc tests were performed to see how arousal varied across conditions and trials. Pairwise comparisons using Dunn‐Bonferroni tests indicated that arousal scores of trials 1 (M = 4.09, Mdn = 4.21, SD = 1.94) and trial 2 (M = 4.29, Mdn = 4.36, SD = 1.79) were observed to be significantly lower from those of trial 3 (M = 5.61, Mdn = 5.90, SD = 2.28) (*p* < 0.05).

**Figure 3:**
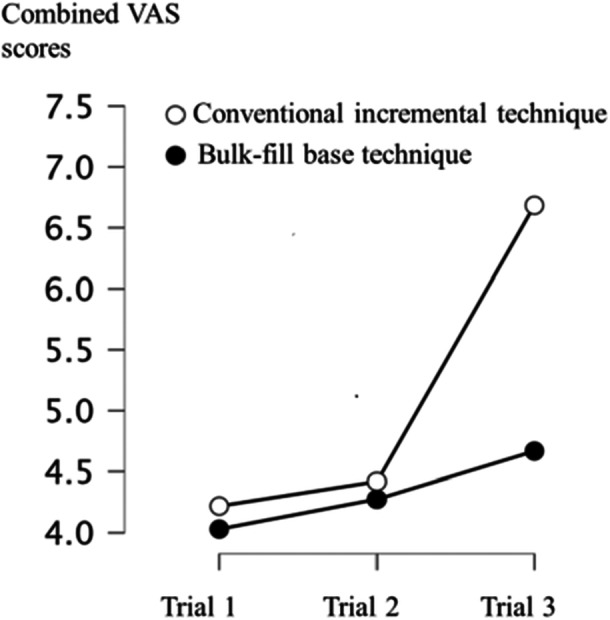
The participant's self‐reported arousal levels (means) using VAS across conditions (plots) and trials (*x* axis).

There was a significant effect of experimental group on the participants arousal levels under time pressure (*F* = 7.78, df = 1.51, *p* < 0.05) (Trial 3; Figure 3). Pairwise comparisons using Dunn‐Bonferroni tests indicated that reported arousal level of trial 3 was significantly higher in the conventional incremental technique group compared to the bulk‐fill base group (*p* < 0.05). Participants using the bulk‐fill base technique reported significantly lower arousal levels (*M* = 4.53, Mdn = 4.20, SD = 2.17) compared to participants using the conventional incremental technique under time pressure (M = 6.68, Mdn = 7.35, SD = 1.86).

Throughout the experiment, a significantly higher mean ranks for G‐force were observed in the conventional incremental technique group compared to the bulk‐fill base group. The Mann–Whitney *U* test was conducted to compare the mean ranks of arousal scores between the conventional incremental group and the bulk‐fill base group. For Trial 2, the arousal scores were higher for the conventional incremental technique group (mean rank = 26.67) than the bulk‐fill base group (mean rank = 16.33); *U* = 112.0, *p* < 0.05. Similarly, for Trial 3, arousal scores in the CIT group (mean rank = 25.71) were higher than in the BF group (mean rank = 17.29); U = 132.0, *p* < 0.05.

Spearman's rank order correlation test revealed a significant positive correlation between G‐force and arousal under time pressure trial (*r*(1) = 0.372, *p* < 0.05).

Hypothesis 2 Time pressure decreases the quality of fillings.

**Figure 4:**
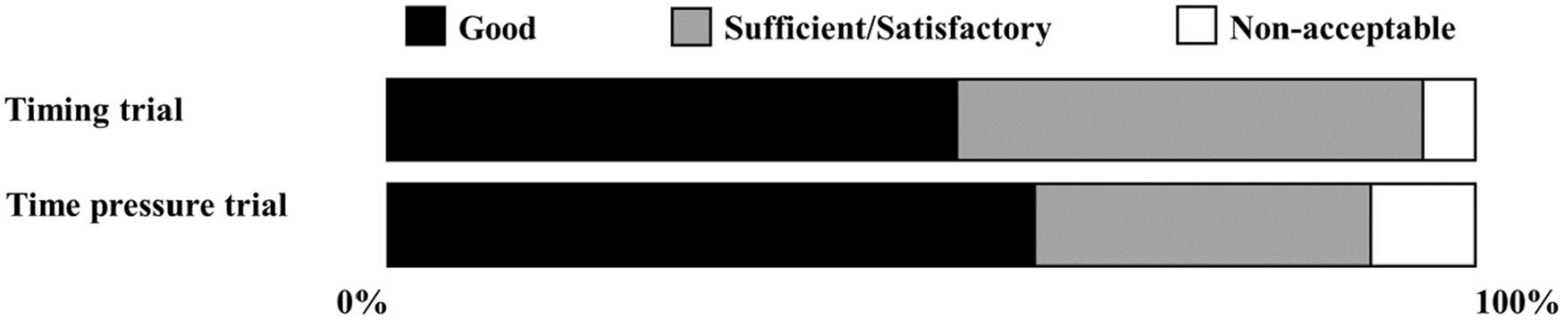
The main effects of time pressure trial on quality evaluations (Trials 2 and 3).

No significant overall effect of the experimental environment on filling quality was observed. (*Χ* ^*2*^ = 2.47, *p* = 0.116, df = 1, Figure 4).

Hypothesis 3 Time pressure has less of an effect on the quality of fillings made using the bulk‐fill base technique than using the conventional incremental technique.

The overall differences in quality between the conventional incremental technique and the bulk‐fill base technique have been published in detail earlier (Leinonen et al. 2023).

**Figure 5:**
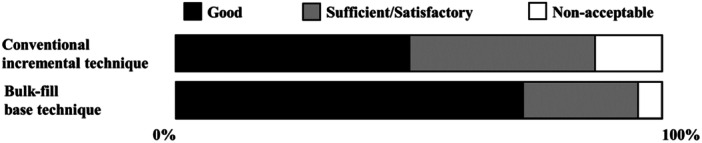
Quality of fillings made under time pressure across techniques (Trial 3).

The proportion of fillings evaluated as good in quality in the time pressure trial was 71.4% for the bulk‐fill base technique and 38.1% for the conventional incremental technique (*Χ* ^*2*^ = 4.71, *p* = 0.030, df = 1, Figure 5). Three fillings placed using the conventional incremental technique and one using the bulk‐fill base technique were evaluated as unacceptable.

Hypothesis 4 Time pressure has more impact on less experienced operators and the quality of their fillings.

During the timing trial and the practice trial the dentists had significantly less arousal compared to the students: Trial 2: (*t* (40) = 3.13, *p* < 0.05; dentist: *M* = 3.39, SD = 1.36; students *M* = 4.98, SD = 1.80; Trial 1: (*t* (40) = 2.78, *p* < 0.05; dentist: *M* = 3.20, SD = 1.20; students *M* = 4.76, SD = 2.13;). But interestingly, the experience of the operator was not associated with the arousal level of the operator under time pressure.

Within the conventional incremental technique group the students had significantly more arousal during the timing trial (*t* (19) = 3.24, *p* < 0.05) and practice trial (*t* (19) = 2.67, *p* < 0.05; dentist: *M* = 3.12, SD = 1.23; students *M* = 5.04, SD = 1.87) compared to the dentists, but no difference was observed during time pressure trial. However, within the bulk‐fill base group no difference in arousal levels between dentists and dental students was observed in any trial.

In the timing trial, the proportion of fillings evaluated as good was 88.9% for dentists and 66.7% for dental students in the bulk‐fill base group. In the conventional incremental group, the proportion of fillings evaluated as good was 33.3% for dentists and 25.0% for dental students. No significant difference of the proportion of fillings classified as good between the dentists and the student was observed for either filling technique (*p* > 0.05).

**Figure 6:**
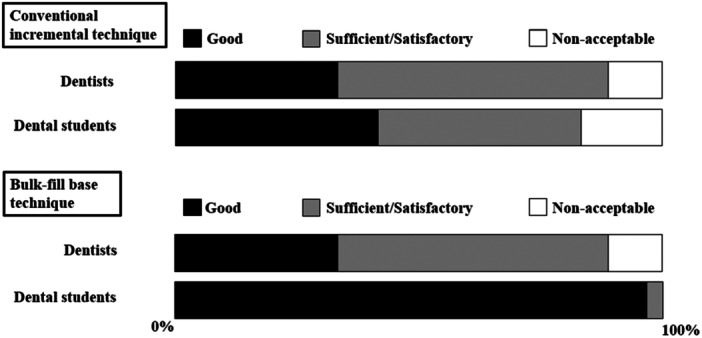
Quality of fillings under time pressure across different techniques based on operator experience (Trial 3).

In the time pressure trial, the proportion of fillings evaluated as good was 44.0% for the dentists and 91.7% for the dental students in the bulk‐fill base group. In the conventional incremental group, the proportion of fillings evaluated as good was 33.3% for the dentists and 41.2% for the dental students. For the bulk‐fill base technique, the proportion of fillings classified as good was significantly larger for the students compared to the dentists (*Χ* ^*2*^ = 5.62, *p* = 0.018, *df* = 1). No corresponding difference was found within the conventional incremental group. In the bulk‐fill base group, the proportion of the fillings evaluated as good increased significantly for the dental students within time pressure (*Χ* ^*2*^ = 6.75, *p* = 0.009, df = 1) (Figure 6).

## 4 Discussion

The main finding of this study was that, under time pressure, employing the bulk‐fill base technique to fill a large class II cavity was associated with a significantly lower arousal level of the operator compared to the conventional incremental technique. Secondly, in terms of quality, the bulk‐fill base technique exhibited significantly better results under time pressure compared to the conventional incremental technique.

In the current study, time pressure had a significantly increasing effect on the participants' arousal levels throughout the experiment. This finding aligns with a previous study on examining bitewing radiograph, where time pressure led to five‐fold stress levels reported by dentists (Plessas et al. 2019). Two‐thirds of general dental practitioners have cited running behind schedule as one of the top ten stressors in their work life (Collin et al. 2019). Additionally, working under constant time pressure have been recognized a source of stress and job dissatisfaction among dentists (Gallagher et al. 2021; Toon et al. 2019).

Stress can affect the performance of a variety of tasks (Baddeley 1972; Gross and Mastenbrook 1980; King et al. 1983), and in the worst‐case scenario, it influences the long‐term well‐being of a person (Yaribeygi et al. 2017). Work‐related stress has been recognized as a significant occupational health risk factor linked to reduced mental well‐being and economic burden to society (Harvey et al. 2017; Hassard et al. 2018). Burnout syndrome is characterized with emotional exhaustion, depersonalization, and reduced personal accomplishment as a consequence to prolonged occupational stress, which can ultimately have negative impact on both dentist and patient safety (Maslach and Jackson 1981; Garcia et al. 2019). Approximately one‐eighth of dentists worldwide have suffered from burnout syndrome (Moro et al. 2022), and it is worth noting that in Denmark, 2.2 dentists per year commit suicide (Hawton et al. 2011).

Interestingly, in our study, participants in the bulk‐fill base technique group reported a 42.9% lower median arousal level under time pressure compared to participants in the conventional incremental technique group. This finding is consistent with a study by Yilmaz et al. (2021), which found that a modern and faster technique was experienced as more comfortable and time‐efficient by dental students and dentists who were already familiar with it. Additionally, we observed a statistically significant increase in the acceleration of hand movements under time pressure, with participants in the conventional incremental group exhibiting significantly higher G‐force compared to the bulk‐fill base group throughout the experiment. Increased arousal, combined with high G‐force, may contribute to less precise work. Findings are noteworthy, particularly given the prevailing reports of burnout and reduced well‐being among dentists and dental students in today's professional landscape (Afrashtehfar and Jurado 2023; Smolana et al. 2022).

The participants reported using the bulk‐fill base and conventional incremental techniques equally often as their usual technique of choice when filling a large Class II cavity. However, nearly 20% fewer dentists than students reported a preference for bulk‐fill base technique. The quality of fillings also appeared unaffected by time pressure or preference for a particular technique; participants who used a filling technique that was not their usual preference did not experience higher arousal or produce fillings of poorer quality compared to those who used their preferred technique.

As indicated by the current results, the bulk‐fill base technique exhibits superior overall quality compared to the conventional incremental filling technique, even under time pressure. Specifically, 71.4% of the fillings made using the bulk‐fill base technique were evaluated as being of good quality under time pressure compared to 38.1% that were of good quality made using the conventional incremental technique.

Surprisingly, within the conventional incremental technique group, the overall quality of the fillings improved by 25% under time pressure compared to the non‐time‐pressured environment. An equivalent number of fillings completed by dentists using the conventional incremental technique were evaluated as good, regardless of the presence of time pressure. Under time pressure conditions, dentists were aware of a specific time limit within which to complete the task. This environment potentially facilitated heightened focus and increased self‐demand, potentially resulting in improved outcomes under more demanding conditions (Baddeley 1972). Conversely, dentists might have been acclimated to working under time pressure due to their clinical experiences and the task was easy enough for them (Mahoney and Chapman 2004; ALQahtani et al. 2016). Additionally, the third round of the experiment may have contributed to familiarization with the environment. It is also noteworthy that the dentist participants were very likely trained in the conventional incremental technique of oblique layering during their studies and are accustomed to utilizing this technique in their clinical work, as reflected in their reported preference. This highlights dentists' well‐established routine and technical proficiency with the conventional incremental technique while also underscoring its inherently challenging nature due to its multiple steps and complexity in all conditions. Taking these factors into account, it can also be questioned whether the time pressure imposed was insufficient for the conventional incremental technique.

In the bulk‐fill base technique group, the quality of fillings deteriorated for dentists due to time pressure, while it improved for students. Only 44% of fillings made by dentists using the bulk‐fill base technique were evaluated as good under time pressure, indicating an almost 50% decrease in the quality of fillings compared to the timing trial. The demand characteristics of the bulk‐fill base technique may have influenced the dentist. Tasks considered easy are performed with little concentration and therefore under‐performed, while tasks considered hard are being performed with high concentration which leads to high‐quality results (Washburn and Putney 2001). It is possible that dentists in the current experiment perceived the bulk‐fill base technique as easy and simple, assuming it did not require advanced skills (Linsenmeier and Brickman 1978). The laboratory conditions might have further affected dentists, as the time pressure on an “easy task” led to it being misinterpreted as unimportant or demanding less concentration. However, the reasons for the reduction in quality are likely not singular. Some dentists may have found the task inherently less interesting or less meaningful, leading to decreased intrinsic motivation to perform well. This reduced motivation may have contributed to a less successful outcome (Baddeley 1972; Daniel and Esser 1980). Additionally, for some dentists, time pressure may have affected their manual ability to complete the task, leading to poorer quality fillings (Baddeley 1972; Gross and Mastenbrook 1980; Szalma et al. 2008).

The bulk‐fill base technique was associated with less arousal under time pressure compared to the conventional incremental technique. However, within the bulk‐fill‐base technique group, an increase in arousal levels was observed under time pressure. This increase in arousal might have led to a decline in performance for the participants, given that optimal performance is often associated with moderate arousal levels, and both excessively high and low arousal levels typically result in decreased performance (Baddeley 1972; King et al. 1983; Yerkes and Dodson 1908). Additional explanatory factors may include the dentists feeling rushed or fatigued during the experiment (considering it was the third round of fillings), as they aimed to complete the task quickly (Baddeley 1972; Daniel and Esser 1980). Alternatively, they might have genuinely been affected by the time pressure, leading to heightened arousal and worsened performance (Szalma et al. 2008).

Time pressure affects people differently. Some individuals might thrive under it, while others experience stress, anxiety, or diminished performance (Rastegary et al. 1993). Dentists are likely routinized to place fillings and probably, at least in routine cases, the procedure can be very automated and intuitive. Under pressure, the reliance on heuristics is likely to increase, but this type of thinking (System 1) is known to be more prone to biases and cognitive errors (Kahneman 2011). In our study, arousal caused by time pressure may have harnessed System 1 thinking leading to poorer quality of fillings. Time pressure may have caused disorientation in the mind of the dentist and increased arousal may have impaired self‐reflection abilities and the performance may have started to deteriorate (Windish 2015). Maybe the dentists got fixated on one phase of the work and things got confused, resulting in failure of the performance (Wickens 2008).

The quality of fillings for dental students improved when moving from the timing trial to the time pressure trial. With bulk‐fill base technique, 25% more of the fillings were evaluated as good after third trial, and with the conventional incremental technique, 16% more fillings were evaluated as good after third trial. The right amount of arousal may have been applied to these students, resulting in good or improved performance (Yerkes and Dodson 1908). Students may have concentrated well and wanted to give their best. Thus, their intrinsic motivation for the task may have been high (Daniel and Esser 1980). Under time pressure, students may have remained in system 2 mode of thinking, engaging their analytical skills to focus intensely on the task rather than rushing through it. This may have led to increase in quality of fillings despite the time pressure (Kahneman 2011). Additionally, the learning curve for students is likely still steep, and even the learning effect of this experimental situation was exploited as the quality improved as the study progressed (Chambers 2012; Yang et al. 2016).

The finding of the current study—that less experienced operators performed significantly better‐quality fillings using the bulk‐fill base technique under time pressure—is somewhat unexpected. To the best of our knowledge, the current study is the first to provide new insights into the effect of experience on the quality of resin composite fillings with and without time pressure. However, the result aligns with another study, with no time pressure though, reporting that dental students achieved significantly higher bond strength compared to dentists (Ueda et al. 2010). Previous research has also shown that experience does not necessarily correlate directly with performance level in dental procedures (Sword et al. 2021; Gomes et al. 2013; Shafiei et al. 2021).

In this study, the bulk‐fill base technique produced a substantial number of fillings of good immediate quality despite the presence of time pressure. Studying dentistry is known to impose significant stress on students (Smolana et al. 2022; Queirolo et al. 2024). The use of the bulk‐fill base technique could potentially expedite procedures in student training clinics (Ahmed et al. 2024), reducing sources of pressure and stress while maintaining high‐quality outcomes. It may also improve learning outcomes and enhance students' enjoyment of their studies. Similarly, dentists could benefit from incorporating the bulk‐fill base technique into their daily practice. Given that time and schedule pressures are recognized contributors to stress and burnout among dentists (Avramova 2023), this technique could alleviate such challenges while ensuring the quality of the fillings.

### 4.1 Limitations

No previously published or pilot data could be used to reliably estimate an appropriate sample size required for this study. It is important to note that the study was conducted in laboratory settings, which should be considered when extrapolating these results to clinical decisions. While efforts were made to replicate clinical conditions within the laboratory, such as using identical teeth with standardized cavities to mimic real‐life large cavities caused by cavitated or symptomatic caries lesions, mounting the teeth on a dental manikin, and involving 42 nonspecialist operators, certain aspects of actual clinical practice, such as patient variability, operator fatigue, and the influence of complex oral environments (e.g. moisture control, salivary flow, and patient cooperation), were not accounted for. These factors may influence the generalizability of the findings to routine clinical decision‐making. Future studies should aim to validate these results in clinical trials conducted under real‐world conditions to better understand their applicability to everyday dental practice.

## 5 Conclusions

Based on the findings of this study, the bulk‐fill base technique shows promise in being resistant to common work environment factors in dentistry, such as time pressure and stress, without compromising the quality of fillings.

## Author Contributions

**Kaisa Leinonen:** conceptualization, investigation, formal analysis, data curation, writing – original draft, visualization. **Jukka Leinonen:** conceptualization, investigation, writing – review and editing. **Mohammed Al‐Haroni:** writing – review and editing. **Jan‐Are Kolset Johnsen:** conceptualization, formal analysis, data curation, writing – review and editing, supervision.

## Ethics Statement

The protocol for this study was approved by the Norwegian Centre for Research Data (reference number 399613). The protocol was reviewed by the Regional Committees for Medical and Health Research Ethics which concluded that the study did not qualify as health research and therefore did not require their approval (reference number 158600).

## Consent

All participants provided written consent to participate in the study.

## Conflicts of Interest

The authors declare no conflicts of interest.

## Data Availability

The authors have nothing to report.
